# Long-term obesity is associated with depression and neuroinflammation

**DOI:** 10.20945/2359-3997000000400

**Published:** 2021-10-29

**Authors:** Fernanda B. Lorena, Bruna P. P do Nascimento, Esther L. R. A. Camargo, Maria M. Bernardi, André R. Fukushima, Julia do N. Panizza, Paula de B. Nogueira, Marllos E. S. Brandão, Miriam O. Ribeiro

**Affiliations:** 1 Universidade Presbiteriana Mackenzie Centro de Ciências Biológicas e da Saúde São Paulo SP Brasil Programa de Distúrbios do Desenvolvimento, Centro de Ciências Biológicas e da Saúde, Universidade Presbiteriana Mackenzie, São Paulo, SP, Brasil; 2 Universidade Federal de São Paulo Medicina Translacional São Paulo SP Brasil Medicina Translacional, Universidade Federal de São Paulo, São Paulo, SP, Brasil.; 3 Faculdade de Ciências da Saúde IGESP Departamento de Pesquisa e Extensão São Paulo SP Brasil Departamento de Pesquisa e Extensão, Faculdade de Ciências da Saúde IGESP, São Paulo, SP, Brasil; 4 Universidade Paulista Instituto de Ciências da Saúde São Paulo SP Brasil Instituto de Ciências da Saúde, Universidade Paulista, São Paulo, SP, Brasil

**Keywords:** Juvenile obesity, behavior, depression, neuroinflammation, cognition

## Abstract

**Objective:**

Obesity is characterized by a state of chronic, low-intensity systemic inflammation frequently associated with insulin resistance and dyslipidemia.

**Materials and methods:**

Given that chronic inflammation has been implicated in the pathogenesis of mood disorders, we investigated if chronic obesity that was initiated early in life – lasting through adulthood – could be more harmful to memory impairment and mood fluctuations such as depression.

**Results:**

Here we show that pre-pubertal male rats (30 days old) treated with a high-fat diet (40%) for 8-months gained ~50% more weight when compared to controls, exhibited depression and anxiety-like behaviors but no memory impairment. The prefrontal cortex of the obese rats exhibited an increase in the expression of genes related to inflammatory response, such as NFKb, MMP9, CCl2, PPARb, and PPARg. There were no alterations in genes known to be related to depression.

**Conclusion:**

Long-lasting obesity with onset in prepuberal age led to depression and neuroinflammation but not to memory impairment.

## INTRODUCTION

It is estimated that more than a third of the world's population is overweight or obese (1). White adipose tissue has long been regarded as an energy storage organ only, but this concept has been modified with the discovery of its ability to also secrete numerous proteins, collectively known as adipokines (2). Although white adipocytes synthesize adiponectin, a protein that increases insulin sensitivity (3), most adipokines are pro-inflammatory, such as leptin (4), resistin (5), *IL-6*, and *TNF-α* (6). The hypertrophic adipose tissue exhibits a dysregulation in the production and secretion of pro- and anti-inflammatory adipokines that leads to a state of chronic, low-grade systemic inflammation related to the pathogenesis of obesity and insulin resistance, hypertension, dyslipidemia, and mood disorders (7,8). Although several other cell types such as macrophages and fibroblasts are found in adipose tissue in addition to adipocytes in individuals who are not overweight, in obesity there is an abundant infiltration in the adipose tissue of macrophages that secrete *TNF-α* and *IL-6*, increasing the systemic inflammation. Thus, the excess of adipose tissue results in a metabolic imbalance that contributes to complications related to obesity.

The cytokines released by both adipose tissue and immune cells can cross the blood-brain barrier (9) and enter the central nervous system (CNS) where they activate the microglia in the CNS, changing their function and morphology, and inducing the secretion of *TNF-α* and *IL-6* (10). Studies show an association between neuroinflammation in the brain and emotional and cognitive abnormalities (11). Increased levels of inflammatory markers are present in depressed patients (12,13). Confirming these findings, it has been shown that the induction of inflammation by peripheral administration of lipopolysaccharides leads to IL1β and *TNFα* release in the brain, inducing depressive behaviors in rodents (14,15).

Adult obesity has been associated with depression (16,17) with a prevalence in obese individuals twice as high as in those of normal weight (16). Mechanisms linking the two disorders include inflammation, oxidative stress, and other endocrine dysfunctions (13). The fact that obese individuals experienced an improvement in their depressive symptoms after diet and weight loss is in line with previous research (18,19). Interconnected limbic brain regions including the ventral tegmental area (VTA), nucleus accumbens (NAc), dorsal striatum, amygdala, hippocampus, and the prefrontal cortex (PFC) are implicated in mediating depression and anxiety (20) and could play a part in these behavior observed in obesity. Corroborating these findings, several studies have shown that inflammatory markers were significantly elevated in the cortex (21,22), striatum (23), NAc (24), and hippocampus (25,26) of obese mice fed a HFD. Another relevant finding is that depressive and anxiety disorders commonly occur together in patients (27,28).

Neuroinflammation is also associated with cognitive impairment. Obese middle-aged adults are at higher risk of cognitive decline (29), particularly on executive function and memory (30). A systematic review revealed a negative association between obesity and cognitive performance at 19-65 years of age (31). However, cross-sectional studies failed to show a higher risk of cognitive decline in late life (29). If in midlife, obesity is associated with an increased risk of cognitive decline, late in life it appears to be neuroprotective (32).

Among prepuberal and puberal children chronic obesity is associated with a high incidence of metabolic alterations (33) and several studies showed deleterious effects on cognition of juvenile obesity (34-37).

Thus, the goals of the present study were to evaluate if chronic obesity initiated in pre-pubertal individuals and lasting until adulthood (38) would lead to cognition impairment and depression in an animal model with obesity induced by a high-fat diet. These are domains known to be affected by multiple systems including thyroid hormones. Indeed, inflammation/cytokines have been shown to regulated T3 production and signaling in the brain. (39-42). Thus, we also looked in the present investigation at genes known to be regulated by T3.

## MATERIALS AND METHODS

All experiments described in the manuscript were conducted in accordance with standards of humane animal care, as outlined in the Ethical Guidelines, and were approved by the Ethics Committee of Mackenzie Presbyterian University (CEUA/UPM n° 112/08/2014).

**Animals:** 4 week-old male Wistar rats were housed in standard plastic cages (40 x 50 x 20 cm) kept at room temperature (22 °C), with a 12-hour dark/light cycle starting at 7:00 am and with access to water and food *ad libitum*. The animals were divided in two groups: Control (n = 10) and Obese (n = 10). Two sets of groups were independently studied.

**Obesity-induced by high-fat diet**: Animals were treated with a high-fat diet (casein, corn starch, lard, cellulose, DL-Methionine, dextrinized starch, choline bitartrate, dibasic calcium phosphate, mineral premix AIN-93G, vitamin premix AIN-93, cheese, and sucrose, resulting in 40.42% fat, 19.9% protein, and 25.67% carbohydrates, totaling 5,7 kcal/g; Rhoster, Sao Paulo, SP, Brazil) for eight months. They were then tested for glucose tolerance and their plasma cholesterol levels were measured (43). The animals continued HFD throughout the behavioral tests, totaling eight months of treatment. Animals from control group were fed with standard diet (whole corn, soy bran, wheat bran, calcium carbonate, dicalcium phosphate, sodium chloride (common salt), vitamin A, vitamin D3, vitamin E, vitamin K3, vitamin B1, vitamin B2, vitamin B6, vitamin B12, niacin, calcium pantothenate, folic acid, biotin, choline chloride, iron sulfate, maganese monoxide, zinc oxide, copper sulfate, calcium iodate, sodium selenite, cobalt sulfate, lysine, methionine, BHT, resulting in 4% fat, 22% protein, and 54% carbohydrates, totaling 1.8 kcal/g; Nuvilab CR1; Nuvital, Brazil). Four weeks after the end of the behavior tests the animals were anesthetized and euthanized by anesthetic excess (Urethane – 3,200 mg/kg) followed by decapitation.

**Glucose tolerance test (GTT):** Following the establishment of the obesity model, basal glucose was measured after a fasting period of 12 hours using a glucometer (One Touch Ultra, Johnson & Johnson, São Paulo, SP). The animals were then given glucose intraperitoneally (2 g/kg BW) and glycemia was measured at 30, 60, 90, and 120 minutes after the glucose dose, as described by Asensio and cols. (2005) (44).

**Plasma triglycerides measurement:** Plasma triglycerides were determined after 12 hours fasting period by colorimetry using commercial kits (Triglycerides liquiform, Labtest, Lagoa Santa, MG), following the manufacturer's instructions and a NanoDrop spectrophotometer.

**Figure 1:**
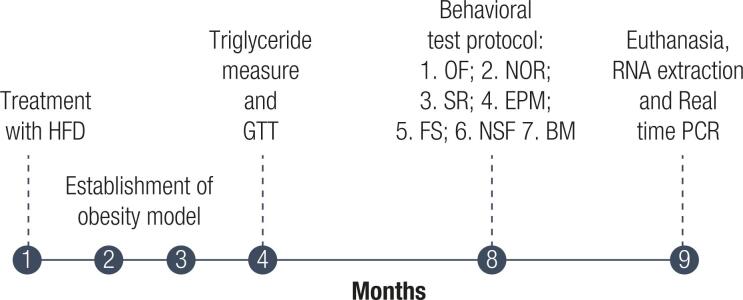
Experimental design.

**Behavioral testing:** All tests were performed between 9 am and 3 pm under dimmed light (15 lux) and were recorded for later analysis by two independent blind observers. The Open Field test was the first one to be performed followed by the Object Recognition test to take advantage of the habituation to the apparatus. Social Recognition, Elevated Plus Maze, Forced Swim, Novelty-suppressed feeding, and Barnes maze tests were performed in this order with a week interval between each one. This was designed to decrease the carryover effect of the tests (Figure 1).

*Open Field test (OF):* The open field test was used to evaluate exploratory activity (45). The animals were placed in the center of a circular acrylic arena (diameter = 100 cm) divided into four central squares and eight peripheral squares (Insight Ltda, Brazil), in a low-light environment (40 Lux) for 10 minutes. Locomotion (total number of lines crossed with all four paws) in the central and peripheral squares was measured. The test was performed for three consecutive times with a 24-hour interval (46).

*Forced Swim Test (FST):* This test was performed to evaluate depressive behavior. The animals were placed in a cylindrical plastic vessel, about 80 cm in height and 40 cm in diameter with water at 32 °C. The test was performed in two stages, training and testing. In the training stage, the animal was placed for about 5 minutes inside the vessel with water for habituation. 24 hours later the animal was placed for the same period inside the vessel with water. The test was recorded for later analysis of (i) swimming and (ii) immobility time (47).

*Elevated Plus Maze test (EPM):* This test was performed to evaluate anxiety and used a maze formed by four, 50 cm long arms (two closed arms and two open arms) suspended 60 cm from the ground (Insight Ltda, Brazil). The animals were placed in the center and remained in the maze for 5 minutes. The test was recorded for later analysis of the time spent in each type of arm (48).

*Novelty-Suppressed Feeding test (NSF):* This test was performed to evaluate anxiety. The animals were fasted for 24 hours prior to the test with free access to water. On the day of the test, they were placed in a square apparatus (43.2 cm x 43.2 cm x 30 cm) with walls and a dark floor. A 20 cm x 20 cm white paper square with 100 grams of food on it was placed in the center of the apparatus, under a strong light (860 lumens). This test evaluated the latency for the animal to start to eat the food, with a time limit of 15 minutes. Immediately after the animal ate the food or when the time limit was reached, the animal was placed in its home cage and the latency to eat the food in the cage was evaluated (49,50).

*Object Recognition test (OR):* This test was performed to evaluate short- and long-term memory and was performed in three stages: training, test, and retest. In the training stage, the animals were placed in the open field arena for 10 minutes with two unknown objects, object A and object B. Three hours later, the test was performed with the animals being placed in the arena for 3 minutes and exposed to object A (known) and object C (an unknown object). The retest was performed 24 hours after the test and the animals were placed in the arena for 3 minutes and exposed to the known object A and object D (a new unknown object). At each stage, the time spent with each object was recorded (51).

*Social recognition test (SR):* This test was performed to assess the animal's memory and was performed in two stages. In the first stage, the animal was placed in the center of a rectangular apparatus (122 x 37 x 78 cm) divided into three equal compartments with an access door into each of the two-lateral compartments. The animal remained in the central compartment with the access doors closed for 10 minutes for habituation in the apparatus. An unknown animal was then placed inside a metal cage (30 x 15 x 19 cm) in the left compartment and a known animal (from the evaluated animal's cage) was placed in the right compartment. The evaluated animal was placed in the central compartment with the access doors open so that it could move freely between the compartments. The test evaluated the time that the animal interacted with the known and unknown animals (52,53).

*Barnes maze test (BM):* This test was performed to evaluate spatial memory. The Barnes maze consists of a white apparatus, formed by a 120 cm diameter circular platform raised 90 cm from the ground with 18, 9 cm diameter holes arranged equidistantly along the perimeter. Only one of the holes gives access to a dark box that contains bedding located under the platform, which is considered the escape box (54). The test was performed in a room with visual cues such as a square or triangle hanging on the white walls in a fixed position to help orientate the animals. Each animal was placed in the center of the maze under a light-proof box and remained there for 30 seconds. The box was then removed, and the animal was observed in the maze for 5 minutes, during which time the animal could find the escape box. Each animal underwent 8 training sessions for four days in the morning and afternoon. On the fifth day, the test was performed and the number of errors until the escape box was found were evaluated.

*mRNA analysis:* Four weeks after the end of the behavior tests five animals randomly chosen were anesthetized and euthanized by anesthetic excess (Urethane – 3,200 mg/kg) followed by decapitation and the whole brain was harvest followed by microdissections and immediately frozen in liquid nitrogen. The PFC of the animals was evaluated for the expression of mRNA of genes related to neuroinflammation, memory, and thyroid hormone regulated genes. The mRNA expression of all genes was evaluated using the real-time PCR technique. Initially, total tissue RNA extraction was performed with TRizol solution (Invitrogen, Carlsbad, CA, USA – 500 μL) and by colorimetric methods using a NanoDrop 2000 spectrophotometer. After extraction, the reverse transcription reaction was performed for complementary DNA synthesis (cDNA), requiring approximately 2-4 μg of the total RNA obtained, processed using the commercial SuperScrit™ First-Strand Synthesis System kit for RT-PCR in Robocycler thermal cycler (Stratagene, La Jolla, CA, USA). Real-time PCR was then performed with the commercial kit QuantiTect™ SYBR^®^ Green PCR (Qiagen, Valencia CA, USA) and the reaction was performed in the StepOne thermocycler (Applied Biosystems, Foster City, CA, USA). Cyclophilin was used as the housekeeping gene for the normalization of the data obtained.

*Statistical analysis:* The experimental data were submitted to statistical analysis to assess their relevance using GraphPad software. The statistical significance of the difference between the mean values was analyzed by the Student's t-test (Body weight gain, GTT, Triglyceridemia, FS, EPM, and NSF) or by two-way ANOVA (OF, OR, SR and BM), followed by the Bonferroni's post-test, with a significance level of p < 0.05.

## RESULTS

**Figure 2:**
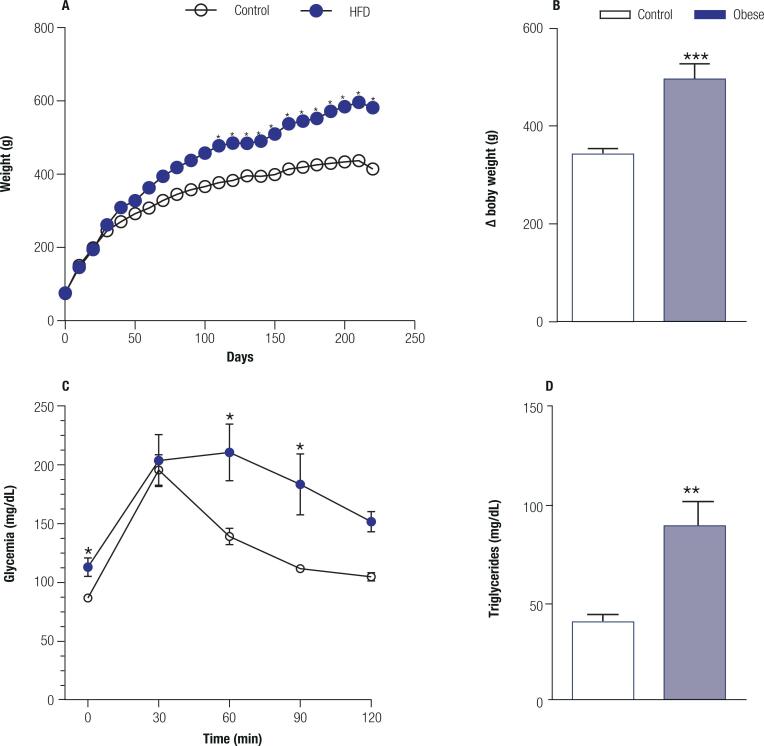
High-fat diet induces metabolic syndrome. **A.** Body weight chart over the 8 months period of treatment with a high-fat diet (40%) in the obese group (n = 10) and a standard diet in the control group (n = 10). **B.** Delta values for body weight over the 8 months of treatment with a high-fat diet (40%) in the obese group and a standard diet in the control group (p = 0.0003). **C.** Glucose tolerance test at the end of the 8 months treatment (p = 0.041) with a high-fat diet (40%) in the obese group and a standard diet in the control group. **D.** Plasma triglycerides at the end of the 8 months treatment with a high-fat diet (40%) in the obese group and standard diet in the control group (p = 0.0045). Values are the mean ± SEM. * p < 0.01 compared with control animals.

**High-fat diet induces metabolic syndrome:** After treatment with a high-fat diet for 8 months, the animals developed a very clear clinical picture of metabolic syndrome. Regarding body weight (Figure 2A), the two-way ANOVA revealed a significant effect of the HFD treatment (F_1, 872_ = 1032; p < 0.0001) and of the time (F_108, 872_ = 71.75; p < 0.001) with interaction between factors (F_108, 872_ = 3.586; p < 0.001). The HFD let to an increase in body of ~50% (t = 4.789; p = 0.0003) (Figure 2B). in the GTT test (Figure 2C), the two-way ANOVA showed a significant effect of the HFD (F_1, 40_ = 23.64; p < 0.0001) and in time (F_4, 40_ = 14.26; p < 0.0001) but without interaction between factors (F_4, 40_ = 1.842; p = 0.139). The treatment with HFD led to a hypertriglyceridemia (t = 3.901; p = 0.0045) (Figure 2D) when compared to control animals.

**Figure 3:**
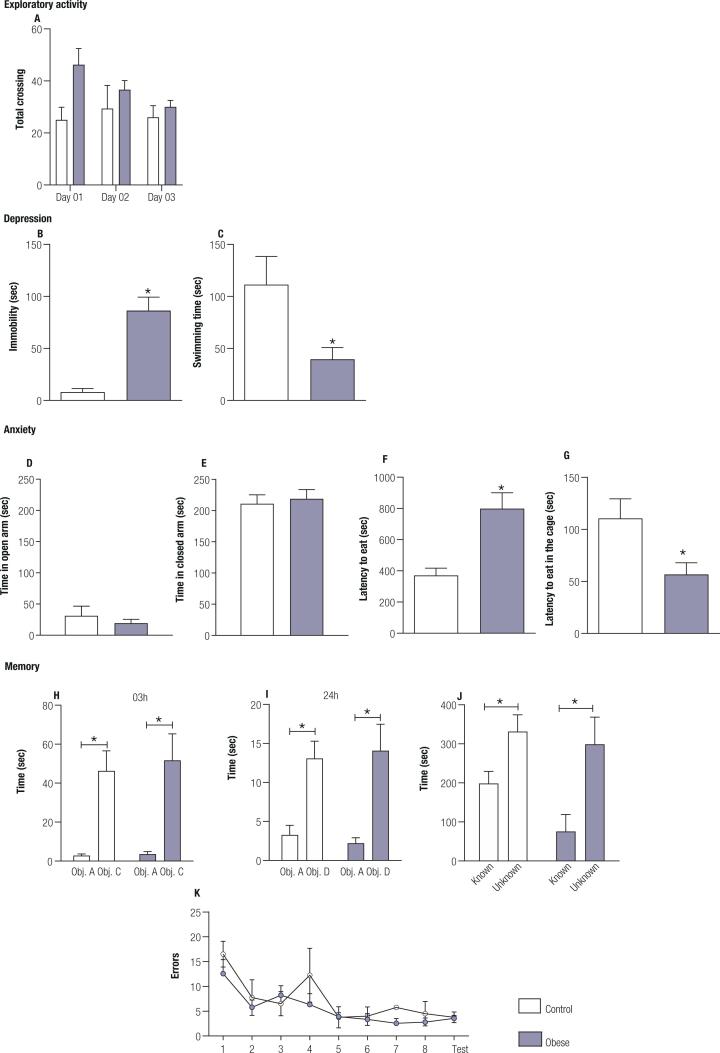
Behavior phenotype in obese animals – Obese (n = 10) and Control animals (n = 10). **A.** Frequency of total crossings in the open field test apparatus on the three days of exposure; **B.** Immobility time in seconds in the forced swim test (p = 0.0025); **C.** Swimming time in seconds of the forced swim test (p=0.0131); **D.** Time in the open arm in seconds in the elevated plus maze; **E.** Time in the closed arms in seconds in the elevated plus maze; **F.** Time in seconds to eat the food in the novelty-suppressed feeding test (p = 0.0172); **G.** Time in seconds to eat in the home cage after novelty-suppressed feeding test (p = 0.0266). **H.** Time in seconds of the animals of each group with each object (Obj. A – known, and Obj. C - unknown) in the object recognition test 3 hrs after the first exposure (p < 0.0001). **I.** Time in seconds of the animals of each group with each object (Obj. A – known and Obj. D – unknown) in the object recognition test 24h after the first exposure (p < 0.0001). **J.** Time in seconds spent by the animals of each group with the known and unknown animal in the social preference test (p = 0.002). **K.** Number of errors in the attempts to find the exit in Barnes maze test. Values are the mean ± SEM.

**Obesity induces depressive-like behavior, but not memory and learning impairment:** To assess the behavioral changes induced by obesity, the animals underwent a series of tests to evaluate exploration, memory, depression, and anxiety. Obese animals presented similar exploratory behavior in the OF apparatus when compared to the control animals. (Figure 3A). Two-way ANOVA showed no significance regarding the HFD treatment (F1, 8 = 2.562; p = 0.148) or time (F1.866, 14.93 = 2.679; p = 0.104) but with an interaction between factors (F2, 16 = 3.756; p = 0.046). Bonferroni's posttest failed to reveal this difference.

Obese animals presented depressive-like behavior as shown by the forced swim test. The data obtained indicated that obese animals spent about 10x more time immobile (t = 3.887; p = 0.0025) (Figure 3B) and swam 3x less (t = 2.955; p = 0.0131) (Figure 3C) than the control animals. Depression is often associated with anxiety, sharing a high rate of comorbidity (55). When subjected to the EPM test, both groups remained in the open (t = 0.7585; p = 0.464) and closed arms (t = 0.381; p = 0.715) for the same time (Figures 3D-E); however, when exposed to a more anxiogenic, the NSF test, the obese animals showed higher latency to eat the food in the apparatus (Figure 3F) (t = 2.825; p=0.017) when compared to the control animals, suggesting an increase in anxiety type behavior. The shorter the time taken to start to eat in the home cage exhibited by the obese animals (t = 2.598; p = 0.026) showed that the delay in eating within the apparatus occurred due to aversive stimulus and not because of an absence of hunger (Figure 3G).

As depression is associated with an impairment in memory processes (56) the cognitive capacity of the animals was assessed using the NOR, SR, and BM tests. Regarding the NOR, the two-way ANOVA test showed at 3h a significant effect of the time (F_1, 22_ = 27.04; p < 0.0001) but not between groups (F_1, 22_ = 1206; p = 0.7317) or for interaction between factors (F_1, 22_ = 0.0705; p = 0.7931). Similar results were observed at 24h with two-way ANOVA test showing a significant effect of the time (F_1, 22_ = 24.01; p < 0.0001) but not between groups (F_1, 22_ = 0.00025; p = 0.9875) or for interaction between factors (F_1, 22_ = 0.2195; p = 0.644). Obese animals recognized the objects to which they were previously exposed, spending significantly more time with the new objects 3h (p < 0.001) (Figure 3H) and 24 h after exposure to the familiar object (p = 0.0014) (Figure 3I). Control animal also recognized new objects 3h (p = 0.0056) and 24h (p = 0.012).

In the SR, the two-way ANOVA showed a significant effect of the time (F_1, 16_ = 13.22; p = 0.0022) but not between groups (F_1, 16_ = 2.527; p = 0.1315) or for interaction between factors (F_1, 16_ = 0.8469; p = 0.3711). The obese animals could discriminate the known animal, spending more time with the unknown animal (p = 0,011) as control (p = 0.011) (Figure 3J).

The two-way ANOVA analysis of the results of the BM test (Figure 3K) showed a significant effect of time (F_8, 99_) = 6.096; p < 0.0001), but no effect between groups (F_1, 99_ = 3.057; p = 0.0835) of interaction between factors (F_8, 99_ = 0.6042; p = 0.7724). These results showed that the obese animals did not present greater difficulties in finding the exit of the apparatus, exhibiting the same performance as the animals in the control group. Taken together, these data show that obesity did not impair the formation and consolidation of memory.

**Figure 4:**
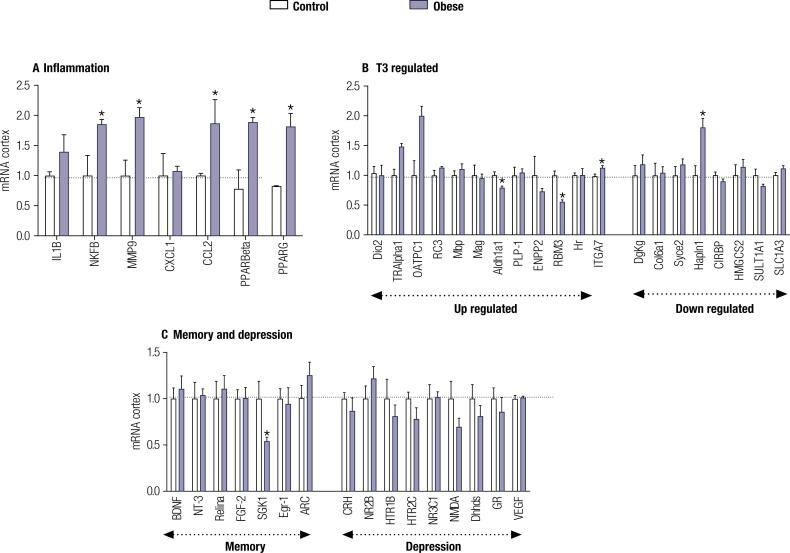
Gene profile in the cortex of obese animals. **A.** mRNA levels of T3-responsive genes. **B.** mRNA levels of inflammation genes and **C.** mRNA levels of genes related to memory and depression, measured by RT-qPCR and using CycloA as the internal control. Values are the mean ± SEM (n = 5) with * p < 0.05, compared with control rats.

**Diet-induced obesity led to neuroinflammation and a minor decrease in T3 signaling in the PFC:** Since obesity is associated with systemic inflammation, we tested if obesity would alter the expression of genes related to inflammation in the PFC, an area known as related to depressive behavior (57,58). A significant increase in the expression of genes involved with inflammation, *NFK*β (t = 2.899; p = 0.03) and *MMP9* (t = 3.429; p = 0.01), and *CCl2* (t = 1.859; p = 0.01) was observed in the PFC of obese animals when compared to control (Figure 4A). An increase in the expression of mRNA for *PPARbeta* (t = 2.768; p = 0,04) and *PPARgama* (t = 2.911; p = 0.02) was also found in the PFC of obese rats.

Considering that inflammation is associated with the modulation of type D2 deiodinase (Dio2) (59,60), we evaluated if the chronic low-grade inflammation would impact the Dio2 mRNA expression and the thyroid hormone-regulated genes in the PFC of the obese rats (Figure 4B). Among the genes studied, a significant reduction in the expression of *Aldh1a1* (t = 3.200; p = 0,01) and *RBM3* (t = 5.637; p = 0,001), positively T3- regulated genes, and a significant increase in the expression of *Hapln1* (t = 3.683; p = 0,01), a negative T3- regulated gene were observed. The other genes remained unchanged (Figure 4B). Notably, the mRNA levels for T3 and T4 transporter, *OATP1C1* (t = 3.390; p = 0,01), and TH receptor, *TRα* (t = 4.168; p = 0,005) and *TR*β*1* (t = 3.045; p = 0,02) were found to be increased in the frontal cortex of obese rats when compared to controls (Figure 4B). The mRNA levels for Dio2 (Figure 4B) were not affected by obesity when compared to controls. Although the obese animals showed marked depression-like behavior, there was no difference in the expression of genes related to depression or memory such as *BDNF* and *NT-3*, which are neurotrophins and mainly associated with increased neurogenesis and cell proliferation (61,62) and *NR2B*, *CRHR2* e *NR3C1*, receptors for glucocorticoids and therefore mediate response to stress (63). We only found a significant decrease in mRNA levels of *SGK-1* in the cortex of obese animals, a gene known to counteract the cortisol-induced reduction in neurogenesis (64) a process associated with depression (Figure 4C).

## DISCUSSION

The present study revealed that chronic obesity with the onset in the prepubertal age induced by a high-fat diet and lasting until adulthood resulted in depressive-like behavior, with no memory impairment. We also showed that obesity was accompanied by a marked increase in the expression of genes involved in the inflammatory response in the PFC. Despite the depressive behavior observed in the obese rats, no changes in the expression of the majority of the genes associated with depression evaluated in our study were observed.

Unexpectedly, obese rats did not exhibit memory impairment. The effect of obesity on cognition is controversial. Several studies show that obesity has a negative impact on memory (65,66), while others showed that memory is not affected by HFD (67) or that HFD can improve memory by increasing the size of the hippocampus (68). In a study performed in adult humans with the onset of obesity in the prepuberal age, the author did not find a reduction in the intelligence scores with the persistence of obesity (69).

The onset of depression may be related to several factors and mechanisms and it is associated with neuroinflammation. Increased secretion of proinflammatory cytokines impairs the metabolism and functionality of neurotransmitters, as well as cerebral plasticity (70). These effects may contribute to mood fluctuations (13) such as higher levels of anxiety (17,71,72), depression (8,73), and cognitive and social deficits (74,75). An increase in expression of the inflammatory genes *NFKβ*, *MMP9*, *CCL2* in the cortex, the region most associated with depression (76), was observed in obese rats (Figure 4A). The increase in the expression of mRNA for *PPARbeta* and *PPARgama* could be due to the inflammatory status in this region (77). These data suggest that obesity-induced systemic inflammation increases the inflammatory response in the brain (78), which may be related to the depressive-like behavior found in our obese animals

Inflammatory cytokines may alter the availability, release, and recapture of monoamines in the brain. The idea that there is a relationship between monoamines and the development of depression is due to the observation of decreased monoaminergic function in the brain of depressed individuals (20,79). Activation of the immune system significantly stimulates the activity of serotonin transporters (SERT), decreasing the availability of serotonin (5-HT), a neurotransmitter associated with the onset of depression (80,81).

The results presented in this study showed that the obese rats spent 10-fold more time immobile in the forced swim test than the control group (Figure 3D-E), which is characteristic of the depressive phenotype (82). Immobility is a behavior related to loss of motivation and may be observed in patients with depression (83). In animal models of depression-like behavior, the immobility observed during the forced swim test is decreased with antidepressants, confirming the idea that immobility can be interpreted as a depressive-like behavior in rodents (47). The possibility that obesity could impair locomotor activity due to weight gain was eliminated by the open field test results because the obese animals showed an increase in exploration of the peripheral and central areas of the open field compared to the control animals as shown by the number of line crossings in the open field (Figures 2A-B), suggesting an increase of exploratory behavior. Depression may also be associated with anxiety and although these two diseases are very different, they share a high rate of comorbidity (55,84,85). Accordingly, the results obtained in the novelty-suppressed feeding test, which evaluated anxiety and anhedonia, showed that the obese animals presented anxious behavior, as they took twice as long to start to eat in the apparatus compared to the animals with a standard diet (Figure 3F). The longer latency to explore the environment (Figure 3F) and, therefore, notice and eat the food in the apparatus also characterized anxiety and anhedonia type behavior in these animals (49). Another possible explanation for the delay in eating the food in the apparatus could be due to changes in cue-triggered food seeking behavior mediated by the mesolimbic dopamine system, a pathway that projects from the VTA to the NAc. High fat diet-induced obesity reduces dopamine levels (86) and type 2 dopamine receptors in the striatum (87,88), which could reduce the interest of the rats to eat the food in the apparatus. However, it is unlikely since when the animals were put back in their home cages, the obese animals took less time to eat (Figure 3G). Thus, it is evident that the obese animals were hungry after being deprived of food and that the delay in eating within the test apparatus occurred because of anxiety and/or depression. It is noticeable that the anxious behavior was observed only in the NSF test that is considered a highly anxiogenic test, but not in the EPM (Figure 3D-E).

Another potential mechanism related to depression is hypo and hyperthyroidism. 1% to 4% of patients with some type of affective disorder also have thyroid dysfunction (89) and treatment with TH may help to reverse the symptoms of depression (90). In patients resistant to treatments with antidepressant drugs, such as selective serotonin reuptake inhibitors, the use of T3 in combination with these drugs has been effective, although the patients are euthyroid (91). Studies performed in humans (92-94) and mice (95) suggest that even subtle changes in TH signaling can have a significant effect on neuronal activity and gene expression. Animal studies have shown an increase in serotonin levels in the cerebral cortex of rats after T3 administration and a decrease in serotonin synthesis in the hypothyroid brain (96-99). However, the data obtained in our study does not support the idea that depression induced by HFD could be related to a decrease in T3 signaling in the brain, since the change in the expression of the genes regulated by T3 was statistically significant but minor. It is also an interesting fact that among the genes often related to depression in the literature, only mRNA levels of the *SGK-1* gene were decreased in the PFC of obese animals (Figure 4B). *SGK1* is a kinase under transcriptional control of various stimuli such as glucocorticoids and has been identified as a mediator of the effects of cortisol on neurogenesis, keeping glucocorticoid receptors active and decreasing hippocampal neurogenesis, which could be of importance for behavioral changes such as depression (64).

Therefore, based on all the results of this study, we can conclude that long-lasting obesity induced by a high-fat diet with the onset in the prepubertal age and lasting until adulthood led to an increase in inflammation in the PFC associated with depression.
